# Neuropathy correlated with imbalanced Foxp3/IL-17 in bone marrow microenvironment of patients with acute myeloid leukemia

**DOI:** 10.18632/oncotarget.8227

**Published:** 2016-03-21

**Authors:** Chen Chen, Yan Liu, Mingqiang Hua, Xiaomei Li, Chunyan Ji, Daoxin Ma

**Affiliations:** ^1^ Department of Hematology, Qilu Hospital, Shandong University, Jinan, China; ^2^ Taian City Central Hospital, Taian, China

**Keywords:** neuropathy, nerve-related molecules, T helper-related molecules, acute myeloid leukemia

## Abstract

Bone marrow (BM) neural tissues are important components of bone marrow microenvironment and play important roles in normal hematopoiesis. Neuropathy of BM can cause immunological alteration in hematopoietic microenvironment. It also can induce the impairment of normal hematopoiesis and promote the development of hematologic diseases. In the present study, we determined the expression levels and clinical significances of nerve-related molecules [nestin, tyrosine hydroxylase (TH), Glial Fibrillary Acidic protein (GFAP) and S100B] and T helper-related molecules (IL-17, Foxp3) in BM of AML patients and controls by immunohistochemical analysis and RT-PCR. Our results showed that the positive rates and expression levels of nestin, TH, GFAP and IL-17 were significantly decreased while Foxp3 and the ratio of Foxp3/IL-17 were statistically elevated in BM of AML patients. We found that there were significantly positive correlations between nestin with TH and IL-17 in BM of AML patients. We also observed significantly negative correlations between nestin with TH and Foxp3/IL-17 ratio. Moreover, the expression of nestin was positively correlated with the overall survival of AML patients. Our study suggests that neuropathy together with imbalanced T helper immunology in bone marrow might play important roles in AML.

## INTRODUCTION

Acute myeloid leukemia (AML) is characterized by malignant clone of hematopoietic stem cells and accumulation of immature myeloblasts in bone marrow. Though the therapeutic strategy has made great progress, there are still many AML patients that fail to achieve complete remission (CR) and relapse at last. Chemotherapy resistance and immune system disorder are the main reasons. Therefore, it is of importance to clarify the pathogenesis of AML and explore novel therapeutic strategy.

Bone marrow microenvironment, playing an important role in the development of leukemia, comprises a rich network of hematopoietic stem cells (HSCs), mesenchymal stem cells (MSCs), osteoblasts, adipocytes, sinusoidal vessels, perivascular reticular cells and bone marrow neural tissue. Bone marrow neural tissue, playing key role in hematopoiesis and immunity, is composed of sympathetic nervous system (SNS) fiber, ensheathing Schwann cells, supporting Schwann cells and nestin+ MSCs [1-3].

Bone marrow hematopoiesis and differentiation are regulated by bone marrow neural tissue. Yamazaki et al described that there were many GFAP^+^ (Glial Fibrillary Acidic protein) Schwann cells in bone marrow. These glial cells ensheath autonomic nerves, express HSC niche factor genes, and are related to HSCs. Moreover, autonomic nerve denervation decreased the number of these nonmyelinating Schwann cells and resulted in rapid loss of HSCs from bone marrow [4]. Méndez-Ferrer et al showed that the sympathetic nervous system directed HSC trafficking by acting on nestin+ niche cells [5]. Another report confirmed chemotherapy-induced neuropathy in the bone marrow was the key factor to prevent hematopoietic reconstruction [6].

Sympathetic nerve injury is closely related to the hematological diseases. Arranz et al described neural alterations arising within the HSPC niche that contribute to MPN (myeloproliferative neoplasm) progression. They indicated that the sympathetic nerve fibers in the perivascular niche were destroyed by MPN cells, which leads to nestin+ MSC apoptosis, HSPC niche alteration, and MPN pathogenesis. Treatment with β3-adrenergic agonists that restored the sympathetic regulation of nestin+ MSCs prevented the loss of these cells. It also blocked MPN progression by indirectly reducing the number of leukemic stem cells [3]. Hanoun et al discovered that neuropathy of the sympathetic nervous system promotes leukemic bone marrow infiltration in an MLL-AF9 AML model [7]. Therefore, the damage of sympathetic nerve in AML bone marrow may be involved in the development of AML. Clarifying its effects and mechanisms is of importance for AML targeted therapy.

Our previous studies showed that Th17 and Tregs were significantly aberrant in patients with AML and other hematological diseases including MDS, ITP and ALL [8-11. Recent researches demonstrated that sympathetic nerve fibers can influence the differentiation of T helper cells through changing the secretion of Th-associated cytokines [12, 13]. Therefore, to elucidate AML pathogenesis and find novel targeted therapy, we detected the expression of nerve-related factors [nestin, tyrosine hydroxylase (TH), GFAP and S100B] in BM of AML patients to explore the role of nerve injury in the development of AML. Furthermore, we assessed the prognostic impact of nerve-related factors expression levels and their association with the T helper-related molecules and clarified their clinical relevance.

## RESULTS

### Nerve-related molecules were down-regulated in bone marrow of AML patients

**Figure 1:**
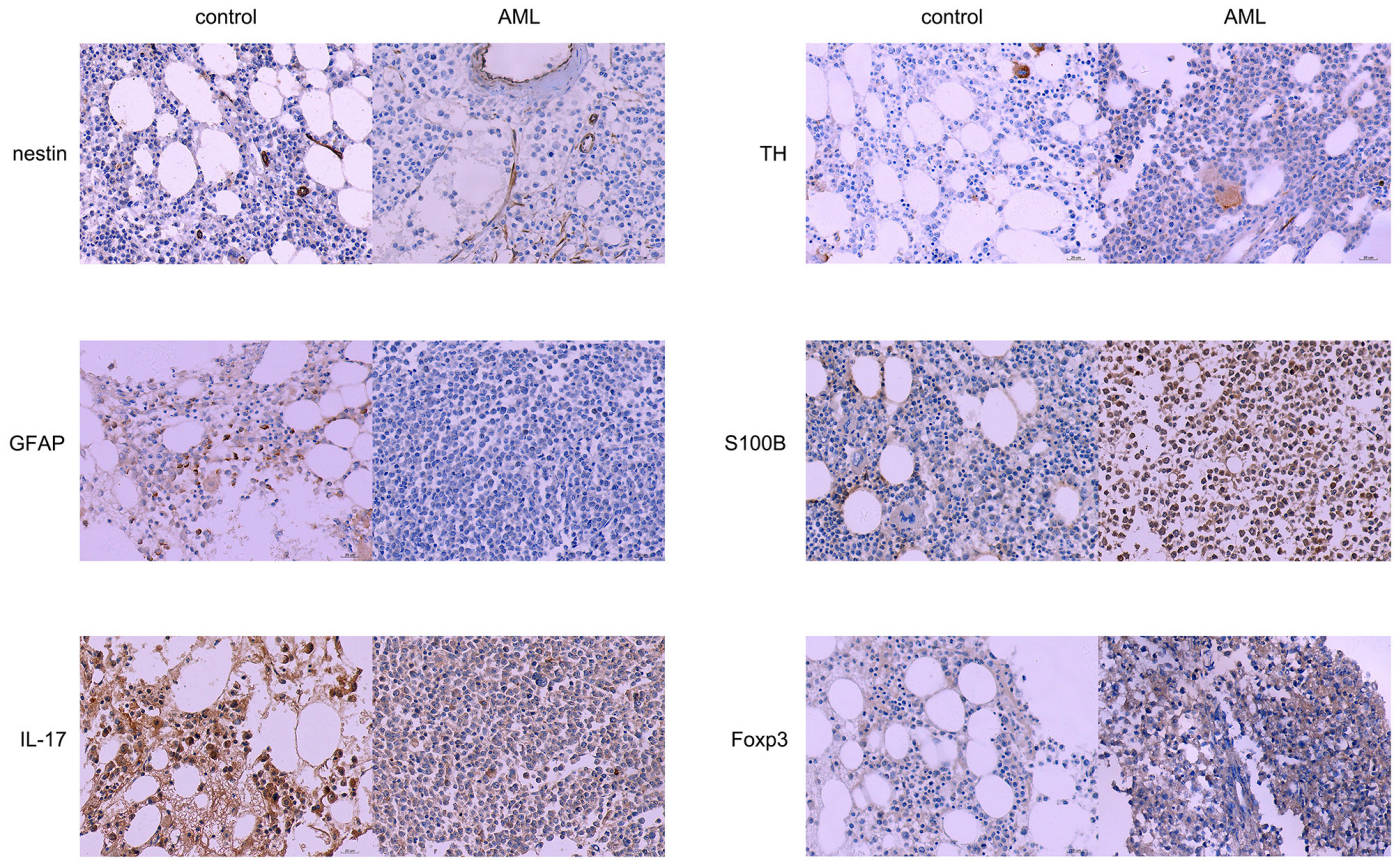
Expression of nerve-and T-helper related molecules in AML patients and controls Sections of bone marrow were stained with antibodies that recognized nestin, TH, GFAP, S100B, IL-17 and Foxp3. Nestin, TH and GFAP proteins were highly expressed in control sections than in AML sections, while S100B and Foxp3 proteins were highly expressed in AML sections than in control sections, × 400.

**Figure 2:**
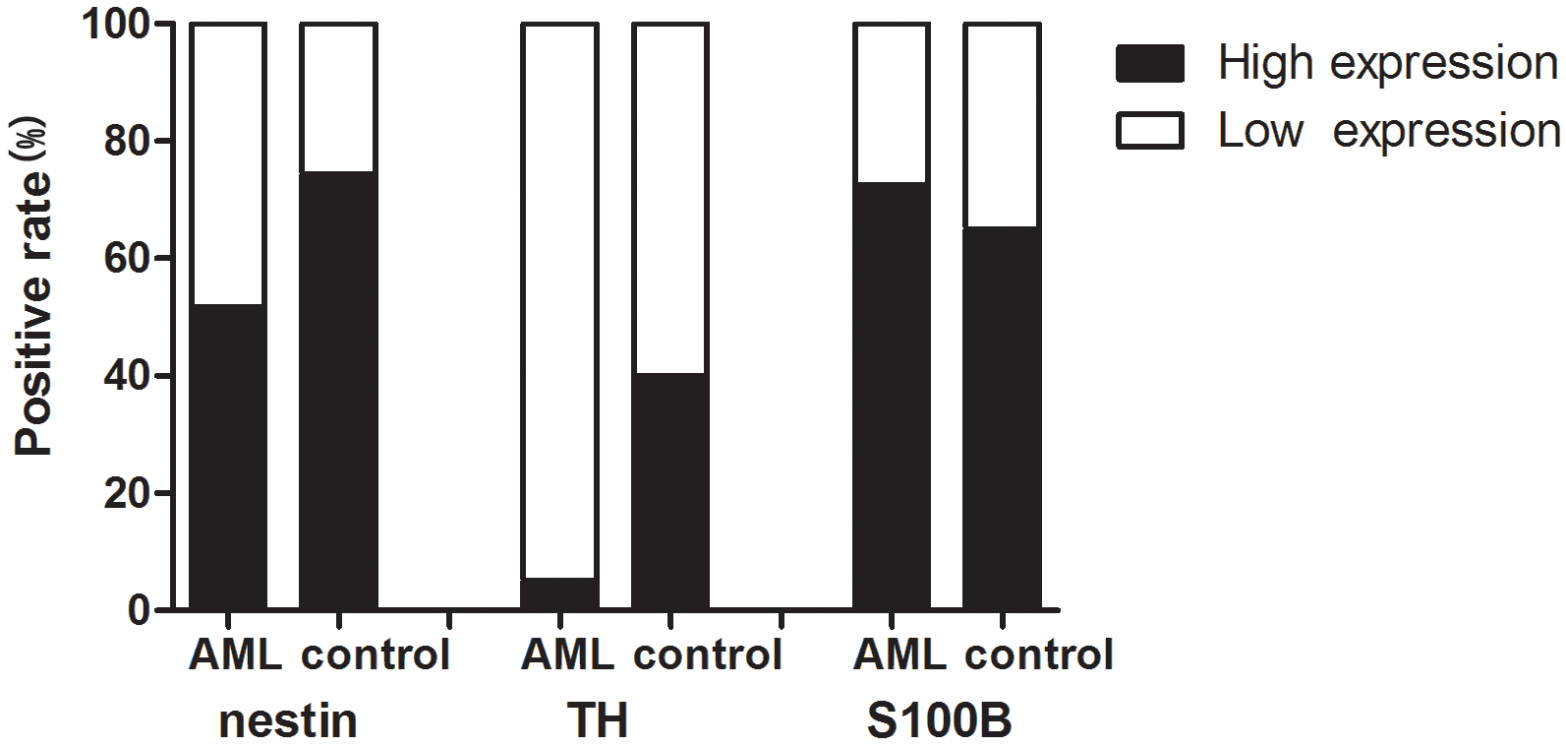
Positive rate of nerve-related molecules expression in AML patients and controls The positive rate of nestin and TH in BM of AML patients was statistically lower than that in controls. No significant difference was found for S100B. Data were analyzed using the Fisher's exact test.

To investigate whether nerve-related molecules (nestin, TH, GFAP and S100B) are involved in BM of AML patients, their expressions were examined using the immunohistochemical staining. Their representative features were shown in Figure 1. The positive rate of high-expression nestin in BM of AML patients (31/60, 51.67%) was significantly lower than that in controls (26/35, 74.29%; P=0.033). The positive rate of TH in BM of AML patients (3/60, 5.00%) was also statistically lower than that in controls (14/35, 40.00%; P=0.000), and we found that most of TH expression was located in megakaryocyte. For the positive rate of S100Bin BM, there was no statistical difference between AML patients (29/40, 72.50%) and controls (13/20, 65.00%; P>0.05) (Figure 2). As for GFAP, We found that there was no positive expression in the AML group (0/40, 0%) and only 2 cases with positive expression (2/20, 10%) in control group.

**Figure 3:**
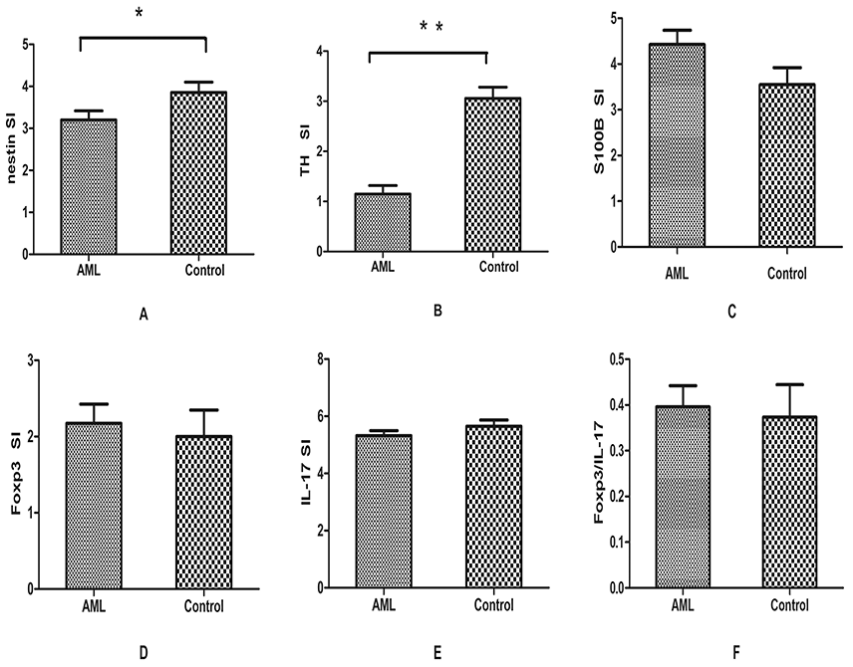
Results of immunohistochemical analysis **A,B.** The expression of nestin and TH was significantly decreased in BM sections of AML compared with controls. **C–F.** No significant difference was found for the expression of S100B, IL-17, Foxp3 and the ratio of Foxp3/IL-17. Data were analyzed using the Student's t-test.

For the immunohistochemical positively-expressed individuals, we further analyzed their expression levels. The expression levels of nestin and TH in positive individuals were significantly decreased in AML patients compared with those in controls (nestin: 3.2±1.68 vs 3.85±1.43, P=0.047; TH: 1.15±1.31 vs 3.05±1.32, P=0.000). (Figure 3A, 3B). There was no statistical difference of expression level of S100B between AML patients and controls (P=0.096) (Figure 3C).

**Figure 4:**
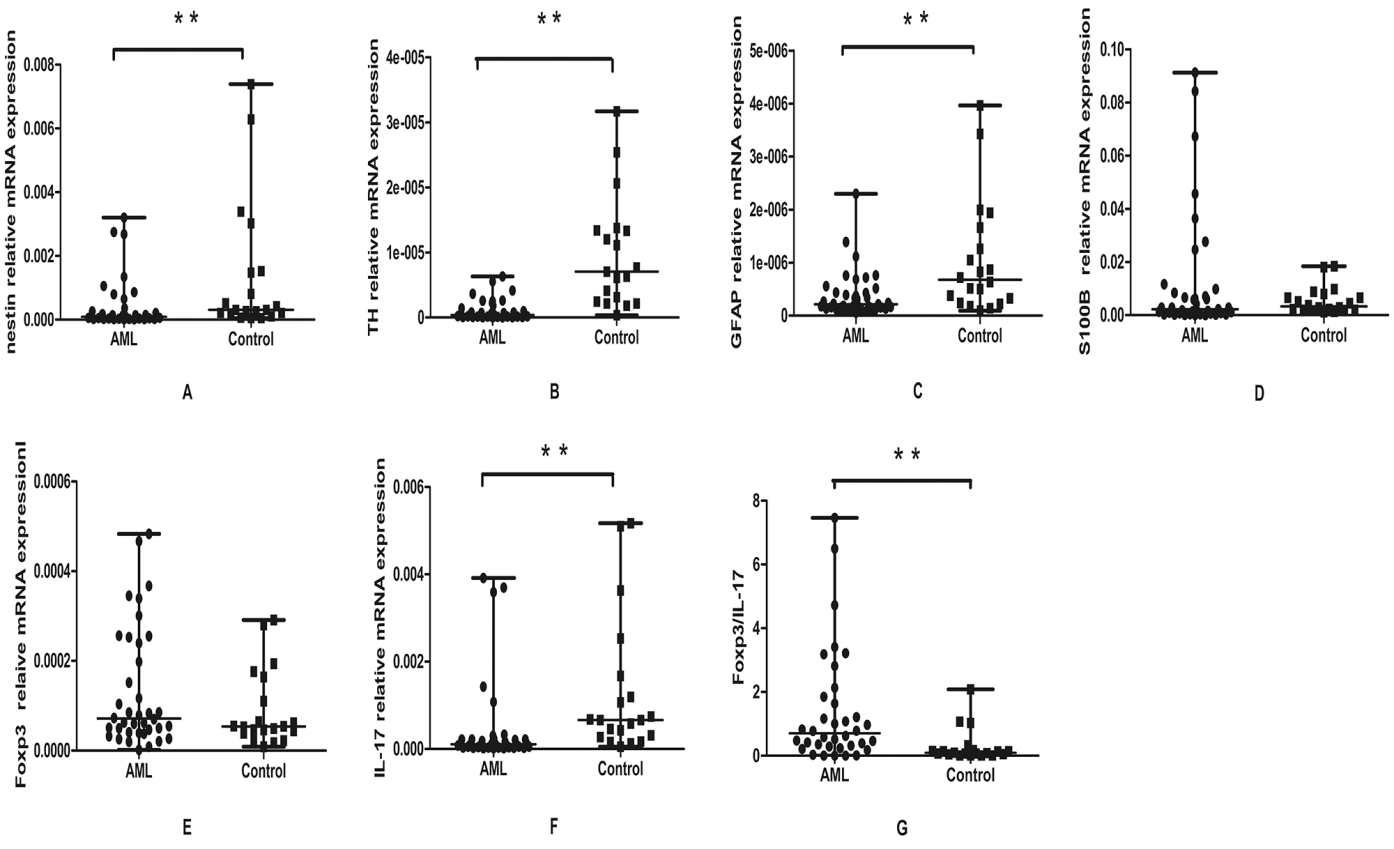
Real-time RT-PCR analysis of nestin, TH, GFAP, S100B, IL-17 and Foxp3 expression in AML patients and controls The mRNA expressions of nestin, TH, GFAP and IL-17 were significantly decreased in AML patients compared to the controls **A,B,C,E.** The expression of S100B was decreased in AML patients and no significance was found **D.** The mRNA expression of Foxp3 was elevated in AML patients compared to the controls, but there was no significant difference **F.** The Foxp3/IL-17 ratio were elevated in AML patients compared to the controls, p=0.000 **G.** Data were analyzed using the Mann-Whitney U test.

To evaluate the mRNA expression of these nerve-related molecules, we also detected them by real-time RT-PCR. Consistent with the immunohistochemical results, we demonstrated that the expression level of nestin was significantly lower in BM of AML patients (median, 8.7×10^−5^; range, 1.2×10^−5^-3.2×10^−3^) than that in controls (median, 3.1×10^−4^; range, 4.7×10^−5^-7.3×10^−3^; P=0.001). The mRNA level of TH in AML patients was also statistically decreased (median, 3.9×10^−7^; range, 0.1×10^−7^-6.3×10^−6^) compared with control group (median, 7.3×10^−6^; range 3.4×10^−7^-3.1×10^−5^; P = 0.00). GFAP was expressed at very low level and its expression level in AML patients (median, 2.2×10^−7^; range, 0.5×10^−7^-2.2×10^−6^) was significantly lower than that in control group (median, 6.7×10^−7^; range, 0.9×10^−7^-3.9×10^−6^; P=0.004). The expression of S100B was decreased in BM of AML patients (median, 2.5×10^−3^; range, 7.6×10^−5^-9.1×10^−2^) compared with controls (median, 3.3×10^−3^; range, 1.1×10^−3^-1.8×10^−2^), however no statistical significance was found (Figure 4A–4D).

### The aberrant expression of T helper-related molecules in bone marrow of AML patients

To evaluate the immunological status in bone marrow microenvironment, Th-related molecules Foxp3 and IL-17 were detected using the immunohistochemical staining. For Foxp3, the highly-expressed positive rate in BM of AML patients (11/40, 27.5%) was higher than that in controls (3/20, 15%). However, no statistical difference between these two groups was observed (P=0.34). As for IL-17, we found it was positive in both AML and control group. Consistently, though the SI score of Foxp3 (2.17±1.58) in AML was higher than that in controls (2.00±1.55), no significant difference was found (P=0.686, Figure 3D). There was no statistical significance of the SI score of IL-17 in AML (5.32±1.09) and control group (5.65±0.98, P=0.27, Figure 3E). The ratio of Foxp3/IL-17 was also analyzed in AML patients and controls. Though the ratio of Foxp3/IL-17 was higher in AML patients than in controls, no significant difference was found (0.39±0.29 vs 0.37±0.31; P=0.79) (Figure 3F).

The mRNA levels of Foxp3 and IL-17 were detected using real-time RT-PCR. Foxp3 expression was seemingly elevated in AML patients (median, range 1.8×10^−6^-4.8×10^−4^) compared with in controls (median, 5.4×10^−5^ range 8.5×10^−6^-2.9×10^−4^) (Figure 4E). However there was no statistical difference between the two groups (P=0.24). The result was in accordance with the immunohistochemical data. As for IL-17, it was shown a significant decrease in AML patients (median, 1.1×10^−4^, range 1.2×10^−5^-3.9×10^−3^) compared with in controls (median, 6.6×10^−4^, range 5.2×10^−5^-5.1×10^−3^; P = 0.00) (Figure 4F). The ratio of Foxp3/IL-17 was also analyzed in AML patients and controls. As shown in Figure 4G, the ratio of Foxp3/IL-17 was significantly increased in AML patients (median, 0.80, range 4.73×10^−4^-7.46) compared with controls (median, 0.08, range 7×10^−3^-2.08; P=0.000).

### The significant correlations were observed between nerve- and T helper-related molecules

**Figure 5:**
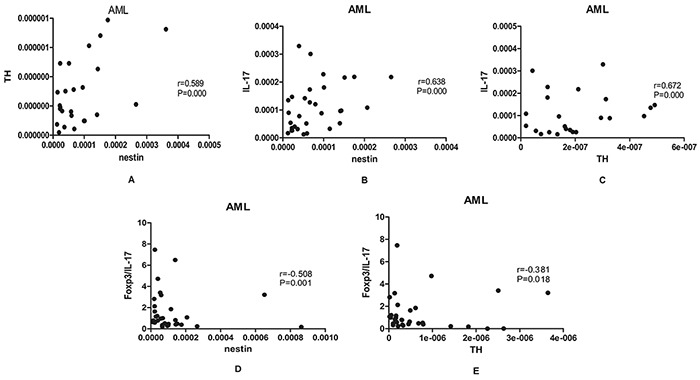
Results of correlations among nerve-related molecules and T helper-related molecules **A,B,C.** A positive correlation was found between nestin and TH (r=0.589, p=0.000), nestin and IL-17 (r=0.638, p=0.000), TH and IL-17 (r =0.672, P=0.000) in BM of AML patients. **D,E.** A negative correlation was found between nestin and Foxp3/IL-17 (r =−0.508, P =0.001), TH and Foxp3/IL-17 (r =−0.381, P=0.018) in BM of AML patients. The spearman correlation test was used for correlation analysis.

To investigate the relationship of nerve-related molecules and T helper cells-related molecules, Spearman correlation coefficients were calculated among these factors. In our results, nestin was positively correlated with TH (r=0.589, p=0.000) and IL-17 (r=0.638, p=0.000) in BM of AML patients. Moreover, a positive correlation was found between TH and IL-17 (r =0.672, P=0.000) (Figure 5A-5C). What's more, we observed a significantly negative correlation between nestin or TH and Foxp3/IL-17 (r =−0.508, P = 0.001; r = −0.381, P = 0.018, respectively) (Figure 5D,5E).

### Clinical relevance of nerve- and T helper-related molecules in AML patients

The relationship between BM levels of nestin, TH, S100B and Foxp3 and clinical features were investigated in AML patients. However, there was no significant correlation in the gender, age, FAB subtypes and the karyotype subgroups (Table 1).

**Table 1:** High-expression of nerve- and T helper -related molecules in different subgroups of AML patients

	n.	nestin	TH	n.	S100B	Foxp3
**Age(years)**						
**<48**	30	14	0	19	13	5
**≥48**	30	17	3	21	16	6
**P-value**		0.6	0.237		0.727	0.237
**Gender**						
**Male**	32	17	3	19	13	4
**Female**	28	14	0	21	16	7
**P-value**		0.507	0.502		0.727	0.488
**FABsubtypes**						
**M1**	1	1	0	0	0	0
**M2**	12	7	2	9	5	2
**M3**	17	7	0	12	11	4
**M4**	6	1	0	5	3	2
**M5**	19	12	1	10	7	2
**M6**	5	2	0	4	4	1
**P-value**		0.552	0.42		0.414	0.907
**Karyotype**						
**Favorable**	22	7	0	13	12	3
**Intermediate**	32	20	2	22	13	7
**Unfavorable**	6	4	1	5	4	1
**P-value**		0.177	0.243		0.096	0.789

Age and gender were analyzed using the Fisher's exact test, and FAB subtypes and karyotype subgroups were analyzed using Chi-Square Tests. FAB, French-American-British classification.

### Nerve-related molecules are associated with the survival of AML patients

**Figure 6:**
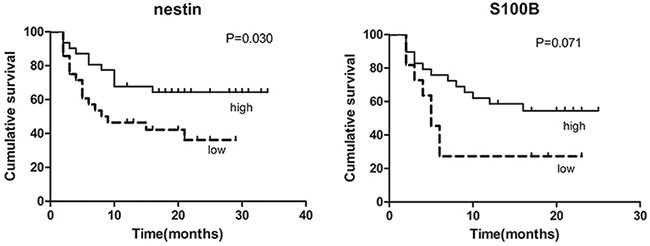
Kaplan-Meier survival plots for overall survival of all patients by the expression of nestin and S100B Data were analyzed using the log-rank test.

We subsequently investigated the correlation between the expression of nerve- or T helper -related molecules and the clinical outcomes of AML patients. The median OS of AML patients was 15.5 months (range, 2.0-34.0 months). Kaplan-Meier curve analysis of the factors associated with OS revealed a significantly longer survival in the patients with higher nestin expression or higher S100B expression. Variables, such as age, gender, TH and IL-17 expression had no impact on the survival. The Kaplan-Meier curves for OS stratified according to nestin and S100B expression in BM of AML are shown in Figure 6. Cox proportional hazards multivariate analysis of the univariate predictors identified that nestin could be a marginal independent prognostic factor (RR, 0.754; 95% CI, 0.561-1.013; P=0.06).

## DISCUSSION

Bone marrow neural tissues are important components of bone marrow microenvironment, which is composed of SNS fibers, nonmyelinating Schwann cells and nestin-expressing perivascular cells containing all bone marrow mesenchymal stem and progenitor cell (MSPC) [14].

Damage of bone marrow neural tissue can cause the alteration of hematopoietic microenvironment, and impair normal hematopoiesis and promote hematologic diseases development. Chemotherapy-induced nerve injury in the mice bone marrow is a crucial lesion impairing hematopoietic regeneration. Neuroprotection is prove to be critical in bone marrow regeneration after genotoxic injury where SNS neuropathy can impair HSC recovery after irradiation or 5-fluorouracilinduced damage [6]. In MPN, the neuroglial damage of BM caused by mutant HSCs is critically contributing to niche damage, which is essential for the development of MPN [3]. Maher Hanoun et al have reported that neuropathy of SNS promotes leukemic bone marrow infiltration in an MLL-AF9 AML mice model [7]. To further investigate the role of BM neuropathy in AML, we detected the nerve-related molecules in myeloid tissue specimens of adult patients with newly-diagnosed AML and control groups, and then observed the alteration of BM nerve tissue.

Nestin, a class VI intermediate filament protein, was originally described as a marker of neuroepithelial stem/progenitor cells in the central nervous system (CNS) [15]. It existed in different cell types in the central and peripheral nervous system and neural crest derivatives [16]. According to recent reports, nestin-positive MSCs are innervated by sympathetic nerve fibers and regulate normal HSCs [5]. In the present study, we demonstrated a notably decreased positive rate as well as expression level of nestin in BM of AML patients compared with the control groups. Moreover, the decreased expression of nestin was positively correlated with poor OS of AML patients. All these results suggest that nestin contribute to the development and prognosis of AML and can be as an important potential therapeutic target.

Tyrosine hydroxylase (TH) is the rate-limiting enzyme of dopamine biosynthesis. It exists in catecholamine neurons and nerve endings within the caudate nucleus, and it specifically labels catecholaminergic fibers[17]. In denervated mice model transplanted with primary human AML cells, the arterioles that remained innervated exhibited significant reductions in the density of ensheathing TH-positive fibers in bone marrow compared to healthy controls [7]. In our study, TH immunohistochemical staining for BM section showed that TH was significantly reduced in BM of AML patients compared with controls. A consistent result was also observed in TH mRNA level. Our results indicated that sympathetic nerve fibers were injured and there was neuropathy in the BM microenvironment of AML patients. Neuroprotection might be considered as a therapeutic method for AML treatment.

Schwann cells in peripheral nervous system are the counterparts of astrocytes in central nervous system. There are two kinds of Schwann cells: myelinating and nonmyelinating. Myelinating Schwann cells which provide the neural myelin sheath express myelin basic protein (MBP), while nonmyelinating Schwann cells ensheathing several small axons express GFAP [4]. Thereby, GFAP is a marker for nonmyelinating Schwann cells. S100B, one member of the calcium-binding protein family, is another marker antigen for Schwann cells [18]. Satoshi Yamazaki found that nonmyelinating Schwann cells are components of the BM niche and maintain HSC hibernation by regulating activation of latent TGF-β [4]. In our results, the mRNA expressions of GFAP and S100B were observably decreased in the BM of AML patients compared with that in controls. Moreover, the mRNA levels of S100B were nearly correlation with OS of AML patients. All these results suggest that Schwann cells were damaged and decreased in AML patients, which may be due to the invasion of immature and dysfunctional leukemia cells. Meanwhile, the damage of nonmyelinating Schwann cells could promote AML progression. Further investigation about the mechanism of neuropathy in AML needs to be clarified.

As for the possible mechanism of neuropathy in AML, previous studies have found that bone marrow nerve injury reduced the expression of IL-1β and TGF-β [3, 19, 20]. And, these cytokines were essential for the differentiation of Th cells including IL-17-positive Th17 and Foxp3-positive Treg subsets. We previously found that Th17 and Treg cells were significantly aberrant in patients with AML and other hematological diseases [11, 21, 22]. Therefore, we speculate that the bone marrow sympathetic nerve may be involved in the development of AML by changing the immune environment, especially the changes of Th17 and Treg subsets and their related cytokines. In this study, we investigated the expression levels of Foxp3 and IL-17 in BM microenvironment of AML patients. Our results showed that mRNA levels of IL17 were significantly decreased in AML patients compared with control groups, while there was no statistical difference for Foxp3 expression. Meanwhile, the imbalanced Foxp3/IL-17 ratio was observed in AML patients. The shift of Foxp3/ IL-17 may synergistically contribute to the suppressive immune system in AML. By correlation analysis, we demonstrated that IL-17 was positively related to nestin and TH in BM microenvironment of AML, and we also observed a significantly negative correlation between nestin and TH with Foxp3/IL-17 ratio. All these suggest that neuropathy and imbalanced Foxp3/IL-17 immunology may contribute together to AML.

In conclusion, our study suggests that neuropathy together with imbalanced T helper immunology in bone marrow might play important roles in AML. Further researches including in vitro or animal model studies are needed to confirm these interesting findings and to expound their detailed roles in the pathobiology of AML.

## MATERIALS AND METHODS

### Patients and controls

A total of 100 patients (54 males and 46 females; age range, 16-79 years) with newly-diagnosed AML were enrolled in this study. AML patients were diagnosed according to French-American-British (FAB) classification system. For the clinical analysis, complete remission (CR), partial remission (PR) and non-remission (NR) were defined according to the criteria of the International Working Group [23]. Chromosome karyotype analysis for newly diagnosed patients was conducted on BM cells after 1-3 days of unstimulated culture and karyotyped according to the International System for Human Cytogenetic Nomenclature (ISCN). Because BM aspiration is a quite invasive procedure, individuals with slight iron deficiency anemia, having no immunological changes, were used as controls. The control group consisted of 55 individuals (22 males and 33 females; age range, 17-78 years). Enrollment occurred between August 2012 and March 2015 in Qilu Hospital, Shandong University (Jinan, China) and Taian City Central Hospital (Taian, China). This study was approved by the Medical Ethical Committee of Qilu Hospital, Shandong University and Taian City Central Hospital. The written informed consent was obtained from all patients before enrollment in the study in accordance with the Declaration of Helsinki.

### Treatment regimen and follow-up

The patients with non-M3 AML subtypes were treated with standard induction chemotherapy (anthracycline and cytarabine). The patients with acute promyelocytic leukemia (APL, subtype M3) received all-trans retinoic acid with or without concurrent induction chemotherapy. After the patients achieved CR, they underwent consolidation chemotherapy with a conventional dose of cytarabine and one anthracycline or with a high dose of cytarabine. The median follow-up duration was 15.5 months.

### Immunohistochemistry

#### Immunohistochemical analysis

Bone marrow biopsy sections from 60 patients and 35 controls were paraffin-embedded prior to immunohistochemical analysis. Characteristics of the patients at the time of sampling were provided in Table 2. Then, the bone marrow sections were deparaffinized in xylene, rehydrated in grade alcohols, and briefly microwaved in citrate buffer to optimize antigen retrieval. Goat serum was blocked for 30 minutes at room temperature. The slides were incubated with primary antibodies raised against human nestin (ab176571, Abcam, USA), GFAP (GA5, Cell Signaling, USA), TH (Tyrosine hydroxylase, MAB318, Millipore, GER), S100B (ab52642, Abcam, USA), IL-17 (ab79056, Abcam, USA) and FOXP3 (ab20034, Abcam, USA), at an assay dependent dilution at 4°C overnight. Phosphate buffered saline was used for all subsequent washes and for antiserum dilution. After extensive washing (3×5 min) to remove excess antibody, the slides were incubated with diluted HRP-labeled goat anti-rabbit antibody (Zhongshan Co. Ltd, Beijing, China) for 1 hour at room temperature. All the slides were then processed by the SP (streptavidin-perosidase) method (Zhongshan Co. Ltd, Beijing, China) for 30 minutes at room temperature. Non-immune IgG was used as negative controls instead of the primary antiserum.

**Table 2:** The characteristics of subjects

	AML patients(n=60)	Controls (n=35)
Age(years)	16-79	17-78
Gender(male/female)	32/28	14/21
WBC(*10/L)	Median:22.1 (0.7-308.5)	Median:4.6 (0.9-12)
HB(g/L)	Median:83 (45-126)	Median:84 (41-137)
PLT(*10/L)	Median:34 (7-266)	Median:188 (72-454)
BM leukemic blast(%)	75 (26-96)	
Karyotype		
Favorable: t(8;21), t(15;17), inv(16)	22	
Intermediate: normal, +8, +22, other	32	
Unfavorable: −7, −5, complex	6	
Median follow up (range, months)	15.5(2-34)	
FAB subtype		
M1	1	
M2	12	
M3	17	
M4	6	
M5	19	
M6	5	

#### Evaluation of immunohistochemistry

For measurement and scoring of each sample, all slides were stained in a single batch and thus received equal staining. Immunostaining of patients and controls was scored visually at 400× by two independent observers using a two-headed microscope. The intensity of stain was scored on a scale of 0-3 as follows: 0, negative; 1, light; 2, moderate; 3, intense. The intensity percentages of positive blasts were measured as the ratio of positive blasts to total blasts. The proportion of positively stained tumor cells was scored as follows: 0 (0-5% positive cells), 1 (6-25% positive cells), 2 (26–50% positive cells), and 3 (>75% positive cells). Staining index (SI) was calculated as the staining intensity score + the proportion score. The SI score of 4 was used to distinguish between low and high expression.

### Real-time reverse transcription-PCR (RT-PCR) analysis

BM samples from all participants were collected into ethylene diamine tetraacetic acid (EDTA)-containing tubes. BM mononuclear cells (BMMCs) were obtained from 40 AML patients and 20 controls using density-gradient centrifugation. Total RNA of these cells was extracted using TRIzol (Invitrogen Life Technologies, Carlsbad, CA, USA), and cDNA was prepared using PrimeScript^TM^ RT Reagent Kit (Takara, Shiga, Japan) according to the manufacturer's instructions. Reverse transcription was performed at 37°C for 15 min, followed by 85°C for 5 sec.

Real-time quantitative PCR (RQ-PCR) was performed using an LC480II Real-time PCR system (Roche, USA) according to the manufacturer's instructions. PCR was performed in a total volume of 10 μl, which included 5 μl SYBR Green real-time PCR mastermix (Toyobo Co. Ltd., Osaka, Japan), 3.2ul DEPC water, 1 μl template cDNA and 0.8 μl forward and reverse primers. The primers of nestin, GFAP, TH, S100B, Foxp3, IL-17 and the endogenous control GAPDH were shown in Table 3. To exclude non-specific amplification and primer-dimer formation, a dissociation curve analysis was performed and PCR products were confirmed by sequencing (Boshang, China). PCR-grade water was used instead of template cDNA for the negative control. The fold-change in the gene expression was determined using the 2^−ΔCT^ method with GAPDH as an endogenous control. All experiments were performed at least twice.

**Table 3:** Primer sequences used for real-time PCR

Gene	Gene bank Accession no.	Sequence
nestin	NM 006617.1	F: CAACAGCGACGGAGGTCTC R:GCCTCTACGCTCTCTTCTTTGA
GFAP	NM 001242376.1	F: GAGTACCAGGACCTGCTCAA R: TTCACCACGATGTTCCTCTT
TH	NM 199293.2	F:GCCCTACCAAGACCAGACGTA R: CGTGAGGCATAGCTCCTGA
S100B	NM 006272.2	F: TGGCCCTCATCGACGTTTTC R:ATGTTCAAAGAACTCGTGGCA
IL17	NM 002190.2	F:CCATAGTGAAGGCAGGAATC R:CGGTTATGGATGTTCAGGTT
Foxp3	NM 014009.3	F: GTGGCCCGGATGTGTGAAG R: GGAGCCCTTGTCGGATGATG
GAPDH	NM 002046.5	F:GCACCGTCAAGGCTGAGAAC R:TGGTGAAGACGCCAGTGGA

### Statistical analysis

Results were expressed as mean ± SD or median (range). Statistical significance between two groups was determined by T Test if the data is normally distributed, while Wilcoxon was used when the data is not normally distributed. Spearman's test was used to evaluate the correlation between the individual expression of the genes studied, and the association of gene expression with clinical features. Furthermore, overall survival (OS) was defined as the number of months from the date of the first diagnosis to death from any cause. Patients were categorized into high and low nerve-related factors expressing subgroups using the SI value 4 as the cut-off. Kaplan-Meier estimation was applied to plot survival curves, and a log-rank test was used to compare survival between groups. Multivariate Cox models estimated the hazard ratio in terms of relative risk (RR) for death, and 95% confidence interval (CI) to determine independent prognostic factors associated with survival. Two-sided P<0.05 were considered statistically significant. All statistical analyses were performed with the SPSS 19 software (SPSS Inc, Chicago, IL, USA).
